# New insights into SUMOylation and NEDDylation in fibrosis

**DOI:** 10.3389/fphar.2024.1476699

**Published:** 2024-12-04

**Authors:** Jin Han, Jun Wu, Wen-Tao Kou, Li-Na Xie, Ya-Li Tang, Da-Long Zhi, Ping Li, Dan-Qian Chen

**Affiliations:** ^1^ Northwest University Chang An Hospital, Faculty of Life Sciences and Medicine, Northwest University, Xi’an, Shaanxi, China; ^2^ Department of Nephrology, Chang An District Hospital, Xi’an, Shaanxi, China; ^3^ School of Pharmacy, Shandong College of Traditional Chinese Medicine, Yantai, Shandong, China; ^4^ Beijing Key Lab for Immune-Mediated Inflammatory Diseases, Institute of Clinical Medical Sciences, China-Japan Friendship Hospital, Beijing, China

**Keywords:** SUMOylation, NEDDylation, fibrosis, ginkgolic acid, MLN4924

## Abstract

Fibrosis is the outcome of any abnormal tissue repair process that results in normal tissue replacement with scar tissue, leading to persistent tissue damage and cellular injury. During the process of fibrosis, many cytokines and chemokines are involved, and their activities are controlled by post-translational modifications, especially SUMOylation and NEDDylation. Both these modifications entail a three-step process of activation, conjugation, and ligation that involves three kinds of enzymes, namely, E1 activating, E2 conjugating, and E3 ligase enzymes. SUMOylation participates in organ fibrosis by modulating FXR, PML, TGF-β receptor I, Sirt3, HIF-1α, and Sirt1, while NEDDylation influences organ fibrosis by regulating cullin3, NIK, SRSF3, and UBE2M. Further investigations exhibit the therapeutic potentials of SUMOylation/NEDDylation activators and inhibitors against organ fibrosis, especially ginkgolic acid in SUMOylation and MLN4924 in NEDDylation. These results demonstrate the therapeutic effects of SUMOylation and NEDDylation against organ fibrosis and highlight their activators as well as inhibitors as potential candidates. In the future, deeper investigations of SUMOylation and NEDDylation are needed to identify novel substrates against organ fibrosis; moreover, clinical investigations are needed to determine the therapeutic effects of their activators and inhibitors that can benefit patients. This review highlights that SUMOylation and NEDDylation function as potential therapeutic targets for organ fibrosis.

## 1 Introduction

Fibrosis is the outcome of abnormal tissue repair processes rather than diseases and has been known to cause persistent tissue damage and cellular injury (Antar et al., 2023; Chen et al., 2018; Taru et al., 2024). Fibrotic tissues are characterized by excessive extracellular matrix deposition and activated fibroblasts accompanied by chronic inflammation (Zhang et al., 2021). Wound healing is effective for repairing injured tissues when the damage is minor or non-repetitive, and only a transient increase in the extracellular matrix and a small amount of activated fibroblasts are observed. However, inflammatory and chronic wound-healing responses are aggravated when the damage is severe, and there is poor elimination of the induced profibrotic factors; in such instances, normal tissue is replaced by scar tissue and often results in organ failure (Henderson et al., 2020; Zhang et al., 2024b). The process of fibrosis begins from injury to the epithelial and/or endothelial cells that release proinflammatory chemokines and profibrotic growth factors; then, macrophages and monocytes are recruited in the injured region and release massive amounts of cytokines and chemokines to induce fibroblast activation. The activated fibroblasts migrate to the injured region and transform into myofibroblasts. Excessive extracellular matrix is also accumulated in such instances, and some parenchymal cells are transformed into fibroblasts or myofibroblasts under stimulation by cytokines and chemokines.

**FIGURE 1:**
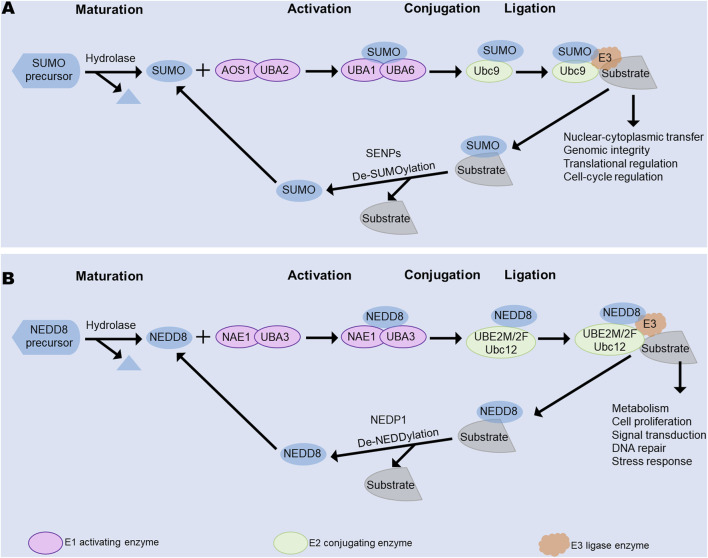
Main processes of SUMOylation and NEDDylation, including activation, conjugation, and ligation. **(A)** Main process of SUMOylation. **(B)** Main process of NEDDylation. Both SUMOylation and NEDDylation share these three processes that are mediated by different E1 activating, E2 conjugating, and E3 ligase enzymes. SENPs facilitate deSUMOylation, while NEDP1 controls deNEDDylation. NAE, NEDD8-activating enzyme; NEDP1, Nedd8 protease 1; SAE1, SUMO-activating enzyme subunit 1; SENPS, sentrin-specific proteases; UBA1, ubiquitin-like modifier activating enzyme 1; UBA2, ubiquitin-like modifier activating enzyme 2; UBA3, ubiquitin-like modifier activating enzyme 3; UBA6, ubiquitin-like modifier activating enzyme 6; UBE2M/2F, ubiquitin-conjugating enzyme E2 M/2F; Ubc9, ubiquitin-conjugating enzyme 9.

During fibrosis formation, many cytokines and chemokines are involved, and their activities are mostly controlled by post-translational modifications (PTMs), including those involving transforming growth factor-β (TGF-β), promyelocytic leukemia (PML), and hypoxia-inducible factor (HIF)-1α (Dai et al., 2020; Lin et al., 2020; Peng et al., 2022). Ubiquitination, phosphorylation, acetylation, and methylation are some of the common PTMs, and numerous studies have confirmed the vital roles of PTMs in fibrosis (Chen et al., 2022; Liessi et al., 2020; Liu et al., 2023c). Notably, some novel PTMs like SUMOylation and NEDDylation are also known to affect fibrosis and have potential as new therapeutic targets against organ fibrosis. Both these modifications entail a three-step process of activation, conjugation, and ligation involving three kinds of enzymes (Figure 1), as will be described in detail in the next section.

In this review, we describe some important cellular and molecular mechanisms of SUMOylation and NEDDylation in organ fibrosis reported over the past 5 years, from their main regulatory enzymes to the processes themselves as well as introduce the roles of SUMOylation, NEDDylation, and their substrates in organ fibrosis. Then, we present the effects of SUMOylation/NEDDylation activators and inhibitors in organ fibrosis. We also discuss the benefits and limitations of SUMOylation/NEDDylation in the treatment of organ fibrosis with the goal of highlighting their therapeutic potentials and clinical treatment.

## 2 PTMs by ubiquitin-like (Ubl) proteins

### 2.1 Ubiquitination

Ubiquitination is a complex enzymatic cascade in which ubiquitin (Ub) units attach to specific residues of a protein, leading to protein degradation, transcriptional regulation, cell survival, protein–protein interactions, and intracellular trafficking. The process of ubiquitination is mediated by three types of enzymes, namely, E1 ubiquitin-activating, E2 ubiquitin-conjugating, and E3 ubiquitin ligase enzymes. First, the free Ub is activated by the E1 ubiquitin-activating enzyme through the participation of ATPs. Ubiquitin conjugates to substrates as a monomer (monoubiquitination) or at multiple sites (multi-monoubiquitination). Then, the activated Ub is transferred from E1 to cysteine at the active site of E2. Finally, the ubiquitinated protein is degraded by the proteasome into amino acids and small peptides or participates in biological processes (Gomarasca et al., 2022; Pellegrino et al., 2022). In this process, depending on E3, ubiquitin is transferred to the substrate via two mechanisms. The really interesting new gene (RING) E3 directly transfers ubiquitin to the substrate, while the homologous to E6AP carboxyl terminus (HECT) E3 or RING-between RING-RING (RBR) E3 transfers ubiquitin to itself and then to the substrate (French et al., 2021). The glycine residue of Ub covalently links to the lysine of the substrate, and Ub also forms Ub chains in this manner. Ub has seven lysine residues, namely, Lys6, Lys11, Lys27, Lys29, Lys33, Lys48, and Lys63. Among these, Lys48-linked polyubiquitin chains are responsible for protein degradation, while Lys29-linked polyubiquitin chains control lysosomal degradation (French et al., 2021). Notably, ubiquitination is a reversible process that is mediated by deubiquitinating enzymes (DUBs) (Liu et al., 2023b). DUBs remove ubiquitin from substrates or ubiquitin chains by reversing the function of the E3 ligase enzyme.

### 2.2 SUMOylation

SUMOylation is an important and reversible PTM similar to ubiquitination; it participates in nuclear–cytoplasmic transfer, genomic integrity, translational regulation, and cell-cycle regulation (Qi et al., 2024; Sun et al., 2024). Small ubiquitin-like modifier (SUMO) proteins are the most well-known Ubls that share a similar three-dimensional structure with Ub. Five SUMO proteins (SUMO1–5) are expressed in mammals, where SUMO2 and SUMO3 are highly similar so as to be called SUMO2/3 (Wang and Matunis, 2023b). SUMO1–3 are widely expressed in tissues, while SUMO4 is mainly expressed in the spleen and kidneys and SUMO5 is mainly expressed in the blood and testes (Gomarasca et al., 2022). Similar to ubiquitination, SUMOylation relies on three classes of enzymes.

**FIGURE 2:**
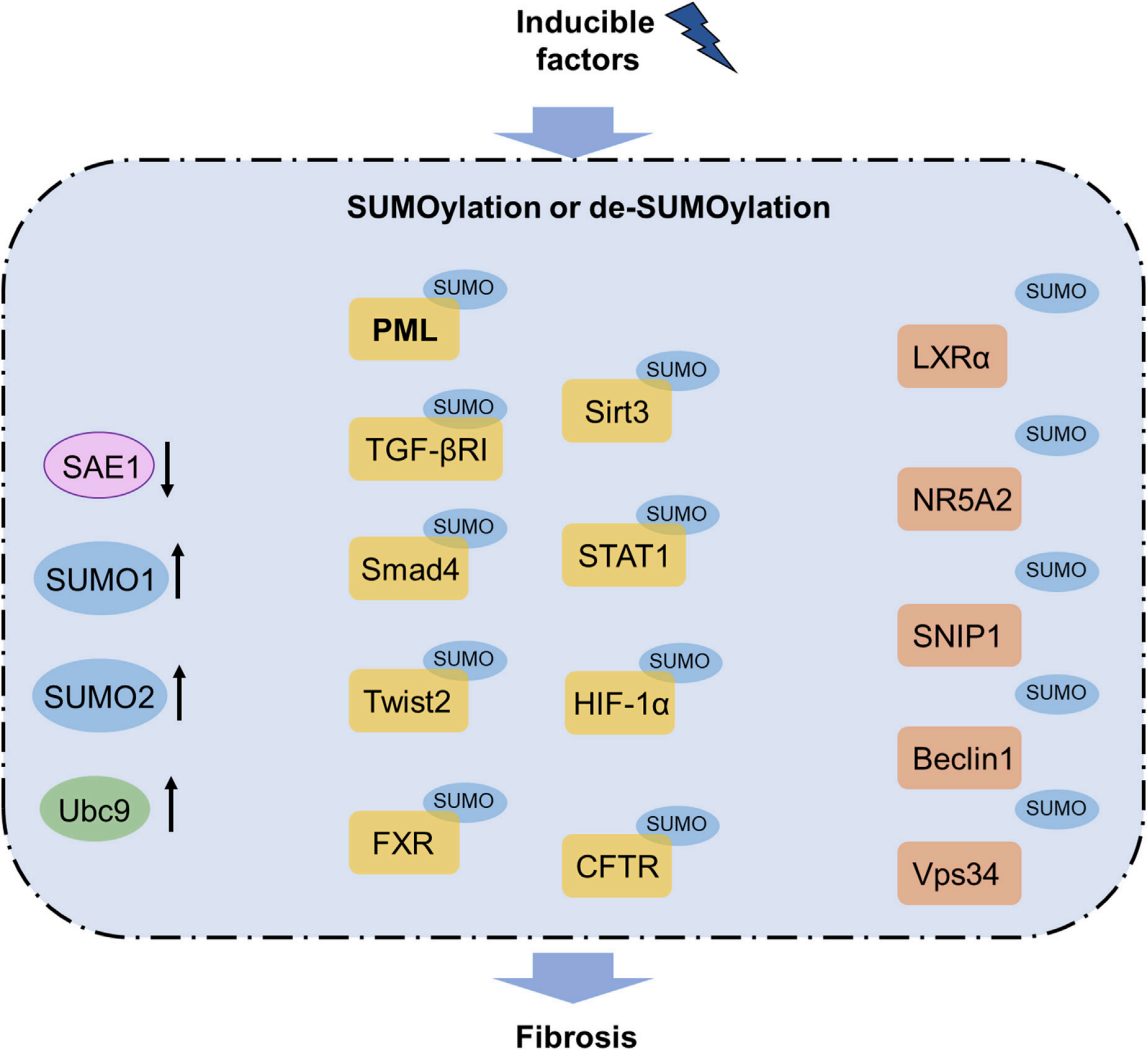
SUMOylation pathway in fibrosis. Increased levels of SUMO1, SUMO2, and Ubc9 as well as decreased level of SAE1 are observed in fibrosis. SUMOylation of PML, TGFβRI, Smad4, Twist2, FXR, Sirt3, STAT1, HIF-1α, and CFTR contribute to fibrosis, whereas SUMOylation of LXRα, NR5A2, SNIP1, Beclin1, and Vps34 can inhibit fibrosis. Notably, SUMOylated PML has been reported to participate in several organ fibrosis and functions as a promising therapeutic target for fibrosis treatment. CFTR, cystic fibrosis transmembrane conductance regulator; FXR, farnesoid X receptor; HIF-1α, hypoxia-inducible factor-1α; LXRα, liver X receptor α; NR5A2, nuclear receptor subfamily 5 group A member 2; PML, promyelocytic leukemia; SAE1, SUMO-activating enzyme subunit 1; SNIP1, Smad nuclear-interacting protein 1; TGFβRI, transforming growth factor-β receptor I; Ubc9, ubiquitin-conjugating enzyme 9.

Before SUMOylation, the SUMO proteins are matured by removing several amino acids using the sentrin-specific protease (SENP) family of proteases. First, the E1 activating enzyme is a heterodimer comprising two SUMO-activating enzyme subunits (SAE1 and SAE2) that activates the SUMO protein at the C-terminal glycine residue to form a thioester bond with SAE2 at the cysteine residue. Then, SUMO is transferred to the only E2 enzyme, ubiquitin-conjugating enzyme 9 (Ubc9), through the formation of a thioester bond during SUMOylation (Zhu et al., 2024). Finally, SUMO is transferred to the substrate at the lysine residue to form a thioester bridge with the glycine residue at the C-terminus of SUMO (Wu and Huang, 2023) (Figure 2). Notably, a specific SUMO consensus motif ΨKXE is identified, where Ψ is a hydrophobic residue while K and E are the respective lysine acceptor and glutamic acid residues, and X is an amino acid. SUMOylation also includes a reversible process called deSUMOylation, in which a SUMO modification is cleaved from a substrate by the SENP family (Pei et al., 2024). The SENP family also mediates SUMO maturation (Brand et al., 2022; Chen et al., 2024a; Wen et al., 2024).

### 2.3 NEDDylation

NEDDylation is a type of PTM characterized by the covalent conjugation of neural-precursor-cell-expressed developmentally downregulated 8 (NEDD8) to a lysine residue in the substrate. NEDDylation plays important roles in metabolism, cell proliferation, signal transduction, DNA repair, and stress responses (Gonzalez-Rellan et al., 2023; He et al., 2023; Lu et al., 2023). NEDD8 is one of the Ubls sharing 80% homology with Ub. NEDDylation involves three enzymatic cascades with NEDD8-activating enzyme (NAE) E1, NEDD8-conjugating enzyme E2 (or ubiquitin-conjugating enzyme E2 M, UBE2M), and substrate-specific NEDD8-E3 ligase.

**FIGURE 3:**
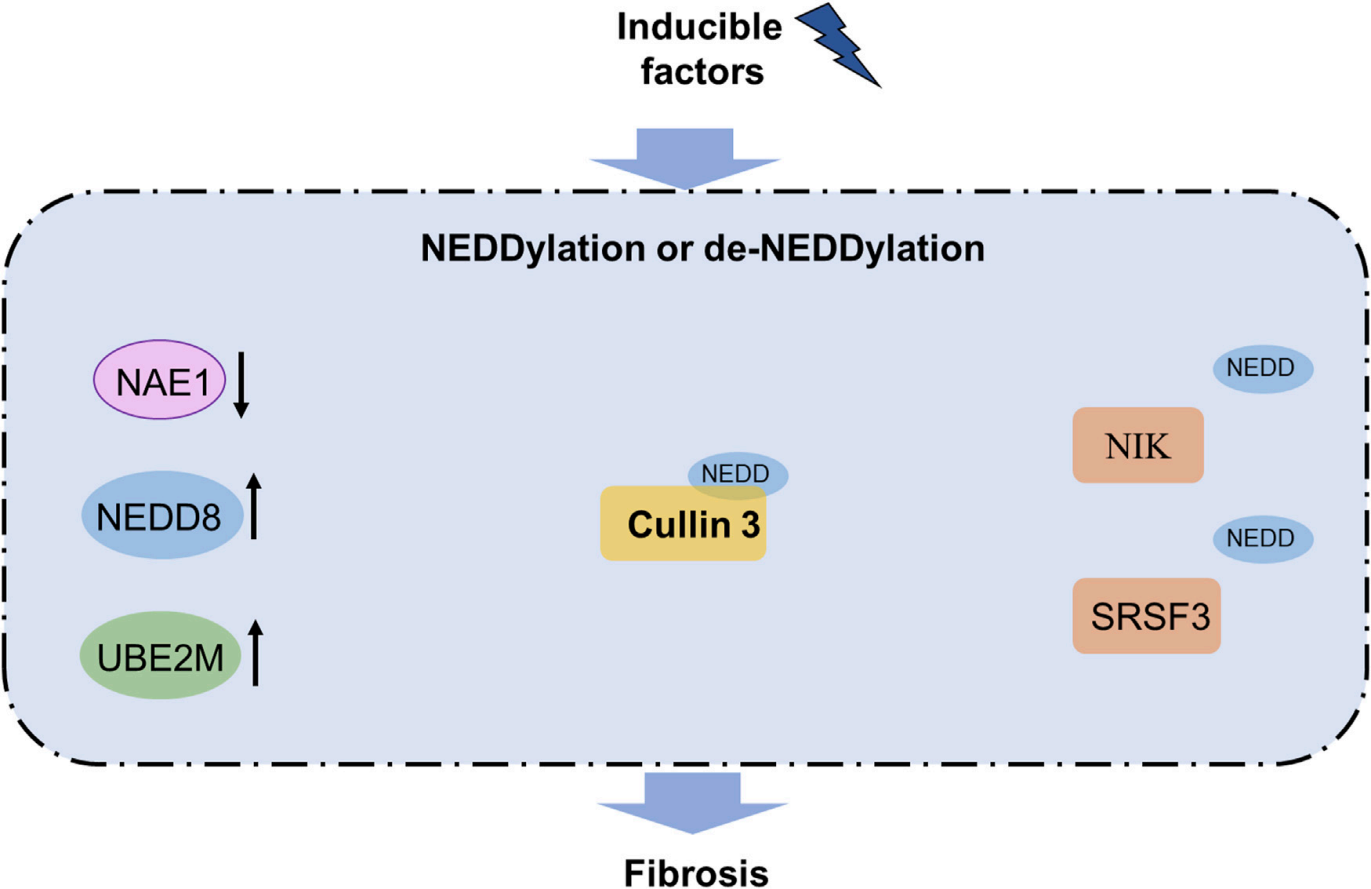
NEDDylation pathway in fibrosis. Increased levels of NEDD8 and UBE2M as well as decreased UBE2M are observed in fibrosis. NEDDylation of cullin3 contributes to fibrosis, while NEDDylation of NIK and SRSF3 can block fibrosis. Notably, NEDDylated PML has been reported to participate in organ fibrosis and functions as a promising therapeutic target for fibrosis treatment. NAE, NEDD8-activating enzyme; NIK, NF-κB-inducing kinase; SRSF3, serine-rich splicing factor 3; UBE2M, ubiquitin-conjugating enzyme E2 M.

Before NEDDylation, the maturation of NEDD8 includes exposure to Gly76 through removal of the C-terminal amino acids from the NEDD8 precursor with a hydrolase such as UCHL3 or DEN1. First, a thioester bond is formed between NEDD8 and the UBA3 subunit of the E1 activating enzyme NAE along with participation of ATPs. Then, NEDD8 is transferred to an E2 conjugating enzyme by a trans-thiolation reaction. Lastly, a substrate-specific E3 ligase contributes to the bond between NEDD8 and the substrate through promotion of the bond between the E2 enzyme and substrate (Figure 3). The most identified NEDDylation substrates are cullins, which are subunits of the cullin-RING E3 ubiquitin ligases (CRLs) (Olaizola et al., 2022; Yu et al., 2022a). Notably, CRLs are common E3 ubiquitin ligases whose activities are facilitated by NEDDylation (Boh et al., 2011; He et al., 2023). In addition, PTEN, p53, and phosphoenolpyruvate carboxykinase 1 (PCK1) are substrates of NEDD8 (Gonzalez-Rellan et al., 2023; Liu et al., 2023a; Xie et al., 2021).

DeNEDDylation involves removal of NEDD8 from a substrate by a NEDD8 isopeptidase (Xiong et al., 2020). Nedd8 protease 1 (NEDP1) is a common NEDD8 isopeptidase that has two kinds of activities; NEDP1 belongs to a small ubiquitin-like-modifier-specific protease family that matures NEDD8 by exposure to the Gly76 residue and removal of the covalent binding between NEDD8 and the substrate (Bailly et al., 2019; Pellegrino et al., 2022).

## 3 Roles of SUMOylation and NEDDylation in fibrosis

### 3.1 Role of SUMOylation in fibrosis

Emerging evidence has revealed the important roles of SUMOylation and deSUMOylation in fibrosis; herein, we describe only a few of the important findings concerning different types of organ fibrosis for the sake of brevity. In the liver, the human PML protein is a key organizer of nuclear bodies that participates in fibrosis. Silencing Ubc9, the only known E2-conjugating enzyme in SUMOylation, alleviates hepatic stellate cell activation, while silencing RNF4, an E3 ubiquitin ligase family member, facilitates the TGF-β/Smad pathway and causes liver fibrosis by enhancing SUMOylated PML accumulation (Dai et al., 2020; Li et al., 2024a). The TGF-β pathway is essential in fibrogenesis, and SUMOylation has been proven as an important target of the TGF-β pathway to treat fibrosis (Ungefroren, 2019). SUMOylation of the TGF-β receptor I occurs at the Lys385 and Lys389 residues, while SUMOylation of Smad4 occurs at Lys113 in the MH1 domain and Lys159 in the linker segment (Wang et al., 2021). Moreover, SAE1 is a promising therapeutic target for suppressing ferroptosis against liver fibrosis by reducing SUMOylation (Zhang et al., 2024a). SUMOylation of the farnesoid X receptor (FXR) occurs at the Lys122, Lys275, and Glu277 residues, and inhibited SUMOylation of FXR promotes its transactivity and suppresses hepatic stellate cell activation against liver fibrosis (Zhou et al., 2020b). The orphan nuclear receptor small heterodimer partner (SHP) alleviates chronic hepatitis C virus (HCV)-induced hepatic fibrosis; SHP regulates gluconeogenesis through Forkhead box O1 acetylation via histone deacetylase 9 (HDAC9) and controls lipogenesis by upregulating the sterol regulatory element binding protein 1c via SUMOylation of the liver X receptor α (Chen et al., 2019). These findings confirm the regulatory effects of SUMOylation in liver fibrosis.

SUMOylation also has notable roles in pulmonary diseases, including hypoxic pulmonary hypertension, idiopathic pulmonary fibrosis, and chronic obstructive pulmonary disease (Zheng et al., 2023) (Table 1). Upregulation of SUMO1 and Ubc9 has been observed in human bronchial epithelial cells after exposure to cigarette smoke extract in chronic obstructive pulmonary disease (Zhou et al., 2020a), and upregulation of SUMO1, SUMO2, and Ubc9 has been noted in the lung tissues of patients with idiopathic pulmonary fibrosis (Yu et al., 2022b). Inhibition of SUMO1 blocks idiopathic pulmonary fibrosis (Yu et al., 2022b), highlighting that SUMOylation has an important role in pulmonary fibrosis.

**TABLE 1:** Profibrotic and antifibrotic effects of SUMOylation and NEDDylation substrates as well as their target organs, related diseases, and biochemical functions.

Target organ	Related disease	Substrates/related proteins	Biological functions	References
SUMOylation				
Liver	Arsenic-trioxide-induced liver fibrosis	PML	Facilitating TGF-β/Smad pathway and accumulating liver fibrosis	Dai et al. (2020), Li et al. (2024a)
	—	TGF-β receptor I at Lys385 and Lys389	Regulating the generation of myofibroblasts and EMTs	Wang et al. (2021)
		Smad4 at Lys 113 and Lys159	Regulating the generation of myofibroblasts and EMTs	Wang et al. (2021)
	Non-alcoholic steatohepatitis (NASH)	FXR at Lys122, Lys275 and Glu277	Suppressing FXR transactivity and hepatic stellate cell activation	Zhou et al. (2020b)
	Thioacetamide-induced liver fibrosis	SAE1 downregulation	Antifibrotic effect	Zhang et al. (2024a)
	Chronic hepatitis C virus-induced liver fibrosis	Liver X receptor α	Regulating lipogenesis and alleviating liver fibrosis	Chen et al. (2019)
	Chronic obstructive pulmonary disease	SUMO1 and Ubc9 upregulation	Profibrotic effect	Zhou et al. (2020a)
Lung	Idiopathic pulmonary fibrosis	SUMO1, SUMO2, and Ubc9 upregulation	Profibrotic effect	Yu et al. (2022b)
	Unilateral ureteric obstruction	NR5A2 at Lys224	Upregulating calreticulin to drive fibrosis	Arvaniti et al. (2016), Politis and Charonis (2022)
Kidney	Folic acid and ischemia–reperfusion-induced acute kidney injury	Sirt3	Suppressing fibroblast-induced repair and promoting fibrosis	Zhu et al. (2023)
	Unilateral ureteric obstruction	HIF-1α	Regulating TGF-β/Smad and NF-κB pathways	Li et al. (2019), Yang et al. (2019)
	Diabetic kidney disease	STAT1	Suppressing STAT1 activity and accelerating EMTs	Gu et al. (2023a)
	Transverse-aortic-constriction-induced cardiac fibrosis	PML	Facilitating PML nuclear body formation and further transforming Pin1 into nuclear to interact with PML, thus promoting fibrosis	Wu et al. (2019)
Heart	Myocardial infarction	PML	Controlling p53 expression to facilitate PML-nuclear-body formation and regulating fibrosis	Huang et al. (2023)
			Promoting p53 recruitment and activation to exacerbate cardiac fibrosis	Qiu et al. (2020)
	Myocardial ischemic injury	Ubc9	Inhibiting apoptosis under oxygen and glucose deprivation against fibrosis	Xiao et al. (2020)
		Vps34 and Beclin1	Facilitating the protein assembly of PI3K-III complexes I and II to inhibit fibrosis	
	Transaortic constriction	Sirt1	Blocking the transformation of cardiac fibroblasts into myofibroblasts to delay fibrosis	Luo et al. (2022)
	Transverse aortic constriction	SUMO2	Dual regulation of SUMO2 and STAT1 to affect fibrosis, hypertrophy, and inflammation	Rangrez et al. (2020)
CFBE41o- airway cells	Cystic fibrosis	CFTR	Modulating biogenesis and degradation	Gong et al. (2019)
Intestine	Crohn’s disease	SNIP1	Inhibiting EMTs and intestinal fibrosis	Chen et al. (2024c)
MCF10A cell	—	Twist2 at Lys129	Accelerating EMTs and promoting mesenchymal phenotypes	Zeng et al. (2021)
NEDDylation				
Liver	CCl_4_-induced liver fibrosis	Global NEDDylation	Promoting the kinase activity of Eph receptor tyrosine kinase EphB1 to trigger fibrosis	Li et al. (2023)
	Bile-duct ligation and CCl_4_-induced liver fibrosis	Global NEDDylation	Promoting chemokine (C-X-C motif) ligand 1 and CCL2 expressions to promote apoptosis and fibrosis	Zubiete-Franco et al. (2017)
	NAFLD	NEDD8	Ameliorating liver fibrosis, lipid peroxidation, lipid accumulation, and inflammation	Serrano-Maciá et al. (2021)
	NASH	Cullin 3	Driving Nrf2 dysfunction and AGER1 downregulation to promote fibrosis	Dehnad et al. (2020)
	Acute liver failure	NIK	Suppressing abnormal NIK activation, aggressive hepatocyte damage, fibrosis, and inflammation	Xu et al. (2022)
	NAFLD and NASH	SRSF3 at Lys11	Promoting SRSF3 degradation and alterations in RNA splicing to alleviate hepatic steatosis, fibrosis, and inflammation	Kumar et al. (2019)
Pancreas	Chronic pancreatitis	UBE2M	Suppressing CCL5 and CD163 expression to drive fibrosis	Lin et al. (2021)
Lung	Cystic fibrosis	NEDD8	Promoting ΔF508-CFTR-induced cystic fibrosis	Ramachandran et al. (2016)

In renal fibrosis, the nuclear receptor subfamily 5 group A member 2 (NR5A2) and Ubc9 are highly expressed in the kidney, while the K224R mutation of SUMOylated NR5A2 fails to upregulate calreticulin to drive fibrosis (Arvaniti et al., 2016; Politis and Charonis, 2022). DeSUMOylation of Sirt3 by SENP1 was found to control macrophage polarization and metabolic stress (Wang et al., 2019; Zhou et al., 2022b). The covalent binding of SUMO1 and Sirt3 increases during acute kidney injury (AKI), and the mutation of lysine to arginine significantly attenuates AKI while minimizing fibroblast-induced repair in a genetically modified mouse model (Zhu et al., 2023). Additionally, renal fibrosis is mainly involved in HIF-1α SUMOylation and SUMO-mediated regulation of the TGF-β/Smad and NF-κB pathways (Li et al., 2019; Yang et al., 2019). STAT1 activation delays epithelial–mesenchymal transitions (EMTs) after high glucose stimulation in the renal tubular epithelial cells, while high glucose levels promote STAT1 SUMOylation to suppress STAT1 activity and accelerate EMT (Gu et al., 2023a). These findings prove that SUMOylation may be a potential therapeutic target against renal fibrosis.

In cardiac fibrosis, obvious TGF-β1, prolyl isomerase NIMA-interacting 1 (Pin1) upregulation, and increased PML SUMOylation have been observed. TGF-β1 stimulation facilitates PML SUMOylation, nuclear body formation, and transformation of Pin1 into the nuclear body to interact with PML (Wu et al., 2019). Another study showed that SUMOylated PML has the ability to control p53 expression as p53 is vital for PML nuclear body formation in cardiac fibroblasts (Huang et al., 2023). The knockout of a poly-SUMO-specific E3 ubiquitin ligase RNF4 has been shown to aggravate interstitial fibrosis and cardiac dysfunction in the animal model of myocardial infarction. RNF4 knockout and PML overexpression facilitate PML SUMOylation as well as p53 recruitment and activation to exacerbate cardiomyocyte apoptosis. The interactions among RNF4, PML, and p53 could be potential therapeutic targets against cardiac fibrosis and apoptosis in myocardial infarction (Qiu et al., 2020). PML overexpression and RNF4 knockdown by small interfering RNA (siRNA) enhance PML SUMOylation, promote p53 recruitment and activation, and exacerbate H_2_O_2_/ATO-induced cardiomyocyte apoptosis, which could be partially reversed by knockdown of p53. Ubc9 has been proven to be a novel therapeutic target for protecting cardiomyocytes from ischemic stress while obviously alleviating cardiomyocyte apoptosis, fibrosis, and improving cardiac function. Ubc9 overexpression attenuates cardiomyocyte apoptosis, while Ubc9 knockout aggravates apoptosis under oxygen and glucose deprivation. A mechanical study showed that Ubc9 promotes SUMOylation of Vps34 and Beclin1, which are two core proteins in the class III phosphatidylinositol 3-kinase (PI3K-III) complex, and facilitates the protein assembly of the PI3K-III complexes I and II (Xiao et al., 2020). Sirt1 SUMOylation blocks the transformation of cardiac fibroblasts into myofibroblasts by suppressing fibrogenesis via the AKT/GSK3β pathway (Luo et al., 2022). The HECT domain containing E3 ubiquitin protein ligase 3 (HectD3) ameliorates pathological hypertrophy, macrophage infiltration, and cardiac fibrosis induced by pressure overload; HectD3 exhibits dual regulation of SUMO2 and STAT1 against hypertrophic and inflammatory effects in cardiomyocytes (Rangrez et al., 2020). These results indicate that SUMOylation is an important tool for treating cardiac fibrosis.

SUMOylation of the cystic fibrosis transmembrane conductance regulator (CFTR) involves its degradation and is a potential therapeutic target in the treatment of cystic fibrosis. Mechanically, the E3 ligase enzyme, which is the protein inhibitor of activated STAT 4 (PIAS4), mediates covalent binding of CFTR to SUMO1 but suppresses such binding to SUMO2/3 (Gong et al., 2019). Other studies have shown that inhibition of SUMOylation can attenuate cystic fibrosis via CFTR (Borgo et al., 2024; Peters et al., 2021), confirming that CFTR is a vital therapeutic target against cystic fibrosis. The long non-coding RNA MSC-AS1 is highly expressed in EMT and intestinal fibrosis through modulation of the Smad nuclear-interacting protein 1 (SNIP1); MSC-AS1 also directly interacts with SENP1 to deSUMOylate and inactivate SNIP1 (Chen et al., 2024c).

Emerging evidence also shows the vital role of SUMOylation in fibrogenesis *in vitro*. Twist2 is a key transcription factor in EMT that contributes to fibrosis. The SUMO2/3-specific E3 ligase zinc finger protein 451 (ZNF451) has been identified as a regulator of Twist2 to maintain its stability. Mechanistic studies show that a direct bond between ZNF451 and Twist2 results in Twist2 SUMOylation at the Lys129 residue and hinders the Ub-dependent degradation of Twist2. Ectopic expression of ZNF451 promotes Twist2 expression and EMT, whereas knockout of ZNF451 inhibits the mesenchymal phenotypes. ZNF functions as a novel mediator in fibrosis by facilitating Twist2 SUMOylation (Zeng et al., 2021). The RAN GTPase-activating protein 1 (RanGAP1) has been identified as a functional partner of SUMOs in fibrogenesis. Mechanically, the RanGAP1-SUMO1 complex mediates nuclear Smad4 accumulation by dissociating Smad4 and CRM1 (Lin et al., 2023). SUMOylation triggers modulation of aldosterone-activated mineralocorticoid receptor transactivation to regulate fibrosis (Gadasheva et al., 2021). The SUMO1-RanGAP1 complex has been proven to be a key molecule for amplification of the TGF-β/Smad and HIF-1 pathways. During fibrogenesis, SUMOylation is activated so that HIF-1α is SUMOylated by SUMO1 at Lys391 and Lys477 (Lin et al., 2020).

### 3.2 Role of NEDDylation in fibrosis

NEDDylation has been proven to be an important regulator of liver fibrosis (Table 1). Hepatic NEDDylation dysfunction causes oxidative stress, inflammation, fibrosis, hepatocyte reprogramming, and liver injury in acute and chronic liver diseases. NEDDylation can be considered a novel therapeutic target against liver fibrosis, and MLN4924 as an inhibitor of NAE shows promising potential in the treatment of liver fibrosis (Xu et al., 2022; Yao et al., 2020). The upregulation of EphB1 is accompanied by increased NEDDylation in activated hepatic stellate cells. This enhanced NEDDylation promotes the kinase activity of the Eph receptor tyrosine kinase EphB1 by preventing its degradation to facilitate proliferation and migration of the hepatic stellate cells. These results indicate that EphB1 could be a promising therapeutic target of liver fibrosis (Li et al., 2023). In liver fibrosis, enhanced NEDDylation is positively related to increased caspase 3 activity to induce hepatic stellate cell apoptosis, whereas inhibited NEDDylation reduces chemokine (C-X-C motif) ligand 1 and C-C motif chemokine ligand 2 (CCL2) expressions to ameliorate apoptosis. Chemokine receptors and cytokines are increased in activated macrophages but decreased in the mouse Kupffer cells after NEDDylation inhibition. These findings indicate that enhanced NEDDylation contributes to liver fibrosis and that NEDDylation may be a promising therapeutic target for treating liver fibrosis (Zubiete-Franco et al., 2017). In the progression of non-alcoholic fatty liver disease (NAFLD) and liver fibrosis, the serum NEDD8 levels are closely related to hepatic NEDDylation. Inhibition of NEDDylation through NEDD8 suppression was shown to ameliorate liver fibrosis, lipid peroxidation, lipid accumulation, and inflammation in the NAFLD mouse model. Deptor is upregulated in NAFLD and liver fibrosis accompanied by suppressed mTOR signaling, increased fatty acid oxidation, and decreased lipid content while its silencing counteracts the antisteatotic effects of NEDDylation inhibition. These results indicate the important roles of Deptor in NEDDylation-inhibition-treated NAFLD and liver fibrosis (Serrano-Maciá et al., 2021). NEDDylation of cullin3 is involved in Nrf2 dysfunction and advanced glycation end product receptor 1 (AGER1) downregulation in liver fibrosis. Overexpression of Nrf2 in the hepatocytes blocks AGER1 decrease and reduces the advanced glycation end-product levels, in addition to suppressing inflammation and fibrosis in the mouse model of non-alcoholic steatohepatitis (NASH) (Dehnad et al., 2020). Dysregulation of NAE1, a regulatory subunit of NAE E1, has been observed in human acute liver failure; loss of NAE in the hepatocytes results in hepatocyte death, inflammation, fibrosis, and eventually liver dysfunction in the mouse model. Notably, NF-κB-inducing kinase (NIK) NEDDylation facilitates its ubiquitination and degradation, whereas inhibition of NIK NEDDylation leads to abnormal NIK activation, aggressive hepatocyte damage, and inflammation in adult male mice with acute liver failure (Xu et al., 2022). Serine-rich splicing factor 3 (SRSF3) modulates liver function, and the loss of SRSF3 deteriorates liver fibrosis and injury. SRSF3 is reduced in human liver samples with NAFLD and NASH along with alterations in the RNA splicing of known SRSF3 target genes. The conjugation of NEDD8 protein with SRSF3 and subsequent proteasome-mediated degradation are induced by palmitic acid. The NEDDylation of SRSF3 occurs at Lys11, and the mutation of SRSF3 (SRSF3-K11R) prevents its degradation and alteration in RNA splicing, which alleviates hepatic steatosis, fibrosis, and inflammation (Kumar et al., 2019).

NEDDylation is involved in fibrogenesis in the lungs, the kidneys, chronic pancreatitis, and cystic fibrosis (Table 1). In pulmonary fibrosis, the cullin-associated and NEDDylation-dissociated 1 (CAND1) level is negatively related to cullin1 NEDDylation in EMT, while the interaction between CAND1 and cullin1 enables the Skp-cullin-F-box protein (SCF) ubiquitin ligase system to boost protein ubiquitination (Zhou et al., 2022c). Familial hyperkalemic hypertension is a monogenic disease caused by mutations in the genes encoding WNK kinases, ubiquitin scaffold protein cullin3, or substrate adapter kelch-like 3 (KLHL3). Compared with wild-type (WT) cullin3, mutant cullin3 Δ403-459 retains the ability to bind and ubiquitylate WNK kinases and KLHL3 while being more NEDDylated and activated. The activated cullin3 Δ403-459 exhausts KLHL3 and prevents WNK degradation, while the loss of cullin3 aggravates FHHt and accelerates renal fibrosis in the murine model (McCormick et al., 2014). Chronic pancreatitis is characterized by irreversible fibrotic and inflammatory disease. Compared with normal healthy controls, UBE2M is remarkably decreased in human chronic pancreatitis tissues accompanied by increased CCL5 and CD163 (markers of M2-type macrophages), indicating the important role of NEDDylation in the pathogenesis of chronic pancreatitis (Lin et al., 2021). In addition, knockout of the ubiquitin ligase SYVN1 or NEDD8 partially restores ΔF508-CFTR-mediated Cl-transport in human cystic fibrosis airway epithelia, indicating the important role of NEDD8 in ΔF508-CFTR-induced cystic fibrosis (Ramachandran et al., 2016). The E3 ubiquitin ligase enzyme Parkin and NEDD4 also have the potential to regulate fibroblast activation during fibrogenesis (Shen et al., 2021).

## 4 Roles of SUMOylation and NEDDylation activators and inhibitors in fibrosis

### 4.1 Roles of SUMOylation activators and inhibitors in fibrosis

Efforts have been made to determine the underlying effects and action mechanisms of SUMOylation inhibitors and activators in organ fibrosis. Recent studies have shown the protective roles of SUMOylation activators against liver fibrosis through activation of SUMOylation (Table 2). Sclareol isolated from *Salvia sclarea* is a potential SUMOylation activator that downregulates SENP1. Treatment with sclareol has been shown to substantially suppress hepatic stellate cell activation, attenuate liver fibrosis, and improve liver function in two mouse models. Mechanistic studies show that sclareol decreases SENP1 expression to inhibit vascular endothelial growth factor receptor 2 (VEGFR2) SUMOylation in LX-2 cells by affecting VEGFR2 intracellular trafficking (Ge et al., 2023). Meanwhile, SUMOylation inhibition attenuates hepatic fibrosis by modulating the profibrotic or antifibrotic factors. Ginkgolic acid is a SUMOylation inhibitor that reduces the expression of SAE1. Mechanically, ginkgolic acid downregulates SAE1 to induce ferroptosis of the hepatic stellate cells, ultimately leading to antihepatic fibrosis effects (Zhang et al., 2024a). FXR is a promising therapeutic target against liver fibrosis whose enhanced SUMOylation weakens the effect of obeticholic acid (FXR receptor agonist) against hepatic stellate cell activation. The triple mutation of FXR at Lys122, Lys275, and Glu277 facilitates its activity. Interestingly, coadministration of obeticholic acid and SUMOylation inhibitors (ginkgolic acid and spectinomycin) has been found to significantly alleviate liver fibrosis (Zhou et al., 2020b). Treatment with S-adenosylmethionine (SAMe), a natural Ubc9-dependent SUMOylation inhibitor, shows obvious hepatic protection through inhibition of hepatic cystogenesis and fibrosis along with decreased liver/body weight ratio and liver volume. Mechanically, SAMe interrupts SUMO1 to suppress proteasome hyperactivity while activating unfolded protein response and stress-related apoptosis (Lee-Law et al., 2021), indicating that it could be a candidate for treating liver fibrosis.

**TABLE 2:** Profibrotic and antifibrotic effects of SUMOylation and NEDDylation activators and inhibitors as well as their target organs, related diseases, and biochemical functions.

Target organs	Related diseases	Compounds	Activators or inhibitors	Target substrates/related proteins	Biological functions	References
SUMOylation						
Liver	Bile-duct ligation and CCl_4_-induced liver fibrosis	Sclareol	Activator	VEGFR2	Decreasing SENP1 expression to inhibit VEGFR2 SUMOylation against fibrosis	Ge et al. (2023)
	CCl_4_ and thioacetamide-induced liver fibrosis	Ginkgolic acid	Inhibitor	SAE1	Downregulating SAE1 to induce ferroptosis of hepatic stellate cells against fibrosis	Zhang et al. (2024a)
	NASH	Ginkgolic acid Spectinomycin	Inhibitor	Global SUMOylation	Modulating STAT3 phosphorylation against fibrosis	Zhou et al. (2020b)
	Polycystic liver diseases	SAMe	Inhibitor	Ubc9	Interrupting SUMO1 to suppress proteasome hyperactivity to activate unfolded protein response and apoptosis against fibrosis	Lee-Law et al. (2021)
Lung	1-NP instillation in lung	1-NP	Activator	ALKBH5	Facilitating ALKBH5 SUMOylation and then causing its ubiquitination and proteasomal degradation to accelerate fibrosis	Li et al. (2024b)
	Idiopathic pulmonary fibrosis	Ginkgolic acid	Inhibitor	SUMO1	Decreasing SUMO1/2/3 and increasing SENP overexpression to suppress Smad4 SUMOylation and regulate EMTs and ROS production against fibrosis	Ding et al. (2022), Yu et al. (2022b)
Heart	Myocardial ischemic Injury	Puerarin	Activator	SUMO2	Facilitating SUMO2 expression and then activating ER/ERK pathway against fibrosis	Zhao et al. (2021)
	Transaortic constriction	(−)-Epicatechin	Activator	Sirt1	Promoting Sirt1 SUMOylation to suppress fibrogenesis via AKT/GSK3β pathway	Luo et al. (2022)
	Transverse aortic constriction	QFYXF	Activator	SERCA2a	Boosting β-arrestin2-mediated SERCA2a SUMOylation and expression	Wang et al. (2024)
	Transverse aortic constriction	LY364947 Juglone	Inhibitor	PML	Reducing the mRNA and protein expression of TGF-β1 and Pin1 to delay cardiac fibrosis process	Wu et al. (2019)
	Myocardial infarction	Ginkgolic acid	Inhibitor	SUMO1	Controlling TGF-β1-induced PML/p53 interaction to suppress cardiac fibrosis	Huang et al. (2023)
	Myocardial ischemic Injury	Arsenic trioxide	Inhibitor	PML	Downregulating RNF4 and PML SUMOylation to suppress myocardial apoptosis and fibrosis	Qiu et al. (2020)
NEDDylation						
Liver	Bile-duct ligation and CCl_4_-induced liver fibrosis	MLN4924	Inhibitor	NAE	Modulating the accumulation of c-Jun against fibrosis	Zubiete-Franco et al. (2017)
	Bleomycin-induced pulmonary fibrosis				suppressing NF-κB responses and MAPK activity against fibrosis	Deng et al. (2017)
	Acute liver failure	N-acetylcysteine	Inhibitor	NAE	Reducing hepatic NAE1 expression to prevent liver inflammation, fibrosis and injury	Xu et al. (2022)
Lung	Human pulmonary fibroblasts	Celastrol	—	Cullin1	Facilitating the interactions between CAND1 and cullin1 to suppress EMTs against fibrosis	Zhou et al. (2022c)
	CCl_4_-induced liver fibrosis	HZX-960	Inhibitor	Cullin3	Blocking the interaction of DCN1 (co-E3 ligase) and Ubc12 and inhibiting cullin3 NEDDylation against liver fibrosis	Zhou et al. (2022a)
Heart	Doxorubicin-induced cardiac fibrosis	MLN4924	Inhibitor	NAE	Maintaining mitochondrial function, alleviating fibrosis, cardiomyocyte apoptosis and oxidative stress damage, and boosting cardiac contractile function	Chen et al. (2024b)
	Pressure overload-cardiac fibrosis	DN-2	Inhibitor	Cullin3	Inhibiting cullin3 NEDDylation to reverse cardiac fibroblast activation	He et al. (2022)
Pancreas	Chronic pancreatitis	MLN4924	Inhibitor	NAE	Promoting CCL5-mediated M2 macrophage infiltration, and the blockage of CCL5 to aggravate fibrosis	Lin et al. (2021)

Exposure to 1-nitropyrene (1-NP) has been found to trigger pulmonary fibrosis in mice, and 1-NP is also identified as an activator of AlkB homolog 5 (ALKBH5) SUMOylation. Mechanically, 1-NP facilitates ALKBH5 SUMOylation followed by ALKBH5 ubiquitination and proteasomal degradation in mouse lung epithelial-12 cells (Li et al., 2024b). Interestingly, inhibition of SUMO1 exhibits protection against pulmonary fibrosis. Ginkgolic acid functions as a SUMO1 inhibitor to block idiopathic pulmonary fibrosis. Mechanically, ginkgolic acid suppresses upregulation of SUMO1/2/3 and promotes SENP overexpression. SENP1 inhibits Smad4 SUMOylation while regulating EMT and reactive oxygen species (ROS) production (Ding et al., 2022; Yu et al., 2022b). These results provide solid evidence that SUMOylation inhibitors and activators are potential candidates against pulmonary fibrosis.

Both SUMOylation inhibitors and activators exert antifibrotic effects in heart tissues (Table 2). Puerarin alleviates cardiac inflammation and cardiac fibrosis by reducing lactate dehydrogenase, COX-2, galectin-3, and cleaved PARP-1. Mechanically, puerarin facilitates SUMO2 expression and SUMOylation before activating the ER/ERK pathway to exert cardioprotective effects (Zhao et al., 2021). (-)-Epicatechin blocks the transformation of cardiac fibroblasts to myofibroblasts against cardiac fibrosis in a Sirt1-dependent manner. The underlying mechanism involves Sirt1 activation by the transcription specificity protein 1 and Sirt1 SUMOylation to suppress fibrogenesis via the AKT/GSK3β pathway (Luo et al., 2022). The Qifu Yixin formula (QFYXF) of traditional Chinese medicine exhibits cardiac protection via restoration of cardiac function as well as amelioration of myocardial fibrosis and hypertrophy. The effects of QFYXF are related to enhanced sarcoplasmic reticulum Ca^2+^-ATPase 2 (SERCA2a) expression and SUMOylation. Molecular docking results show that the main active compounds in QFYXF have high affinities to β-arrestin2, SERCA2a, and SUMO1, with SERCA2a having high affinity to SUMO1. QFYXF exerts antifibrotic effects by boosting β-arrestin2-mediated SERCA2a SUMOylation and expression (Wang et al., 2024). Furthermore, inhibition of PML SUMOylation by LY364947 or Juglone significantly reduces the mRNA and protein expressions of TGF-β1 and Pin1 to delay cardiac fibrosis (Wu et al., 2019). Pharmacological inhibition of the SUMO pathway by the SUMO1 inhibitor ginkgolic acid can substantially control TGF-β1-induced PML/p53 interactions to suppress cardiac fibrosis (Huang et al., 2023). Treatment with arsenic trioxide, which is an ROS inhibitor, reduces RNF4 expression and PML SUMOylation to suppress myocardial apoptosis and fibrosis against myocardial infarction (Qiu et al., 2020). Various natural products have also been identified as vital regulators in SUMOylation (Liu et al., 2022). These findings provide potential candidates against cardiac fibrosis through the regulation of SUMOylation.

### 4.2 Roles of NEDDylation activators and inhibitors in fibrosis

NEDDylation activators and inhibitors have also been found to modulate liver fibrosis (Table 2). MLN4924, also called as pevonedistat, is a first-in-class NAE inhibitor. The inhibition of NEDDylation by MLN4924 triggers hepatic stellate cell apoptosis to prevent liver injury, inflammation, and fibrosis through the accumulation of c-Jun (Zubiete-Franco et al., 2017). MLN4924 controls the NEDDylation of CRLs to delay liver fibrosis progression by suppressing NF-κB responses and MAPK activity (Deng et al., 2017). Treatment with N-acetylcysteine, a glutathione surrogate and antioxidant, has been found to significantly reduce hepatic NAE1 expression to prevent liver inflammation, fibrosis, and injury in the acute liver failure mouse model (Xu et al., 2022).

Celastrol is a pentacyclic triterpene compound isolated from *Tripterygium wilfordii* as a novel treatment for pulmonary fibrosis; it exhibits antifibrotic effects through the covalent linkage of CAND1 at the Cys264 residue. Celastrol treatment influences cullin1 NEDDylation; celastrol also exerts antifibrotic effects in a CAND1-dependent manner and facilitates interactions between CAND1 and cullin1 to activate the Skp1/cullin1/F-box ubiquitin ligases that control EMTs (Zhou et al., 2022c). Additionally, HZX-960 has been identified as an inhibitor that blocks the interaction of DCN1 (co-E3 ligase) with Ubc12 and inhibits cullin3 NEDDylation against liver fibrosis. HZX-960 attenuates liver fibrotic signaling by suppressing collagen I and α-SMA while promoting Nrf2, HO-1, and NQO-1, indicating that it is a promising therapeutic candidate against liver fibrosis (Zhou et al., 2022a).

In the heart, MLN4924 exerts antifibrotic properties (Table 2); MLN4924 mitigates doxorubicin-induced cardiotoxicity by maintaining mitochondrial function, alleviating cardiomyocyte apoptosis, suppressing oxidative-stress-induced damage, boosting cardiac contractile function, inhibiting cardiac fibrosis, and impeding cardiac remodeling. Mechanistically, MLN4924 delays cardiac NEDDylation and offers cardiac protection by limiting NAE activity (Chen et al., 2024b). The antifibrotic effects of DCN1 have also been demonstrated in cardiac fibrosis, where DCN1 is upregulated in the cardiac fibroblast and pressure overloaded mouse hearts. The compound DN-2 has been optimized as a potent DCN1-Ubc12 inhibitor and shown to have high affinity to DCN1; DN-2 effectively reverses cardiac fibroblast activation by inhibiting cullin3 NEDDylation (He et al., 2022). These results highlight the potential of DCN1 as a promising therapeutic target against organ fibrosis. The inhibition of global NEDDylation by MLN4924 obviously aggravates chronic pancreatitis by promoting CCL5-mediated M2 macrophage infiltration, and the blockage of CCL5 counteracts MLN4924-mediated chronic pancreatitis. A mechanistic study showed that inactivation of CRLs stabilizes the level of HIF-1α to facilitate CCL5 upregulation and transactivation (Lin et al., 2021).

## 5 Conclusion and perspectives

PTMs enhance the functional diversity of proteins by modulating the covalent modifications of the functional groups or proteins to induce slicing or degradation, thereby influencing the physiological and pathophysiological processes (Schepers et al., 2023; Wang and Tong, 2023c). Ubiquitination controls protein degradation, transcriptional regulation, cell survival, protein–protein interactions, and intracellular trafficking (González et al., 2023; Gu et al., 2023b). The three-step process of ubiquitination involves activation, conjugation, and ligation through the E1 activating, E2 conjugating, and E3 ligase enzymes. The processes of SUMOylation and NEDDylation are also similar to ubiquitination, but their enzymes are distinguishable from ubiquitination. In SUMOylation, the E1 activating enzyme consists of the SAE1 and SAE2 subunits, and Ubc9 is the only E2 enzyme. In NEDDylation, the NAE E1 activating enzyme consists of the NAE1 and Uba3 subunits. SUMOylation participates in nuclear–cytoplasmic transfer, genomic integrity, translational regulation, and cell-cycle regulation, while NEDDylation contributes to DNA replication and repair, chromatin structure, translational regulation, and caryomitosis (Ren et al., 2024; Tan et al., 2023; Xu et al., 2023; Zou et al., 2023). SUMOylation and NEDDylation can be reversed by SENPs and NEDP1, respectively.

Emerging evidence has shown that SUMOylation and NEDDylation play pivotal and diverse roles in organ fibrosis by mediating the PTMs of profibrotic or antifibrotic factors. In the liver, SUMOylation of FXR at Lys122, Lys275, and Glu277 along with the liver X receptor α has been found to attenuate fibrosis (Chen et al., 2019; Zhou et al., 2020b) while SAE1 contributes to fibrosis. Upregulation of SUMO1, SUMO2, and Ubc9 have been reported to aggravate pulmonary fibrosis (Yu et al., 2022b; Zhou et al., 2020a). In the kidneys, SUMOylation of NR5A2 at Lys224 and that of STAT1 have been found to promote EMT and fibrosis (Arvaniti et al., 2016; Gu et al., 2023a; Politis and Charonis, 2022), whereas SUMOylation of Sirt3 has been shown to suppress fibroblast-induced repair and fibrosis (Zhu et al., 2023). SUMOylation of HIF-1α is also involved in renal fibrosis through regulation of the TGF-β/Smad pathway (Li et al., 2019; Yang et al., 2019). SUMOylation of Vps34, Beclin1, and Sirt1 have been noted to obviously suppress cardiac fibrosis (Luo et al., 2022; Xiao et al., 2020), while SUMO2 was observed to affect cardiac fibrosis through dual regulation along with STAT1 (Rangrez et al., 2020). Additionally, SUMOylation of CFTR and SNIP1 was found to facilitate fibrosis (Chen et al., 2024c; Gong et al., 2019), while SUMOylation of Twist2 at Lys129 was noted to accelerate fibrosis *in vitro* (Zeng et al., 2021).

Notably, although the roles of SUMOylation and NEDDylation are diverse in different organs, their regulation of PML, HIF-1α, and TGF-β are common in fibrogenesis. SUMOylation of PML promotes fibrosis in the lung and heart tissues, and the underlying mechanism is involved in facilitating PML nuclear body activation of the TGF-β/Smad pathway as well as recruitment and activation of p53 (Dai et al., 2020; Huang et al., 2023; Li et al., 2024a; Qiu et al., 2020; Wu et al., 2019). Further investigations have highlighted the vital role of SUMOylation in the TGF-β/Smad pathway; SUMOylation of the TGF-β receptor I at Lys385 and Lys389 as well as Smad4 at Lys113 and Lys159 can control the generation of myofibroblasts and EMTs to influence hepatic fibrosis (Wang et al., 2021). Ubc9 was found to participate in pulmonary and cardiac fibrosis; upregulation of Ubc9 was observed in fibrotic pulmonary tissues, whereas overexpression of Ubc9 was noted to suppress cardiomyocyte apoptosis against fibrosis (Xiao et al., 2020; Zhou et al., 2020a). These studies have proved the vital role of SUMOylation in organ fibrosis and its function as a potential target against organ fibrosis.

Global NEDDylation was found to exacerbate liver fibrosis through activation of the Eph receptor tyrosine kinase EphB1 as well as upregulation of the chemokine (C-X-C motif) ligand 1 and CCL2 expression to accelerate fibrosis (Li et al., 2023; Zubiete-Franco et al., 2017). NEDDylation of cullin3 induces Nrf2 dysfunction and AGER1 downregulation to trigger fibrosis (Dehnad et al., 2020; Kumar et al., 2019), and NEDDylation of NIK SRSF3 at Lys11 can alleviate liver fibrosis (Xu et al., 2022). UBE2M has been found to drive fibrogenesis in chronic pancreatitis by suppressing CCL5 and CD163 expressions (Lin et al., 2021). Interestingly, NEDD8 ameliorates liver fibrosis but promotes cystic fibrosis in the lungs (Ramachandran et al., 2016; Serrano-Maciá et al., 2021), indicating the diverse roles of NEDDylation in different organ fibrosis. These results provide solid evidence and highlight NEDDylation as a potential therapeutic target for treating organ fibrosis.

The activators and inhibitors of SUMOylation are potential candidates that can influence organ fibrosis. Sclareol activates VEGFR2 SUMOylation against hepatic fibrosis, and SAMe inhibits Ubc9 to interrupt SUMO1 and activate unfolded protein responses against hepatic fibrosis (Ge et al., 2023; Lee-Law et al., 2021). The SUMOylation activator 1-NP promotes ALKBH5 SUMOylation and subsequent ubiquitination as well as proteasomal degradation to trigger lung fibrosis (Li et al., 2024b). In the heart, puerarin functions as a SUMOylation activator to facilitate SUMO2 expression and activate the ER/ERK pathway against fibrosis (Zhao et al., 2021). (-)-Epicatechin promotes Sirt1 SUMOylation to suppress cardiac fibrogenesis by modulating the AKT/GSK3β pathway (Luo et al., 2022). QFYXF promotes SERCA2a SUMOylation and expression in the treatment of cardiac fibrosis (Wang et al., 2024), while LY364947 and juglone obviously suppress PML SUMOylation to reduce the mRNA and protein expressions of TGF-β1 and Pin1 to delay cardiac fibrosis (Wu et al., 2019). Arsenic trioxide helps PML SUMOylation to reduce RNF4 against myocardial apoptosis and fibrosis (Qiu et al., 2020). Notably, ginkgolic acid alleviates fibrosis in the liver, lungs, and heart by downregulating SAE1, modulating STAT3 phosphorylation, and influencing PML/p53 interactions (Ding et al., 2022; Huang et al., 2023; Yu et al., 2022b; Zhang et al., 2024a; Zhou et al., 2020b). MLN4924 as a first-line NEDDylation inhibitor exhibits antifibrotic properties in the liver, heart, and pancreas. MLN4924 exerts antifibrotic effects by modulating c-Jun accumulation, NF-κB responses and MAPK activity, mitochondrial functions, and CCL5-mediated M2 macrophage infiltration (Deng et al., 2017; Lin et al., 2021; Zubiete-Franco et al., 2017). N-acetylcysteine reduces hepatic NAE1 expression to prevent hepatic inflammation and fibrotic injury (Xu et al., 2022), while celastrol targets cullin1 to facilitate the interactions between CAND1 and cullin1 to suppress EMTs and pulmonary fibrosis (Zhou et al., 2022a). Both DN-2 and HZX-960 inhibit cullin3 NEDDylation against fibrosis; DN-2 inhibits cullin3 NEDDylation to reverse cardiac fibroblast activation, while HZX-960 targets cullin3 to block the interaction of DCN1 (co-E3 ligase) and Ubc12 as well as inhibit cullin3 NEDDylation against liver fibrosis (Zhou et al., 2022c). Even though they target different profibrotic/antifibrotic factors, the activators and inhibitors of SUMOylation/NEDDylation exhibit therapeutic properties against organ fibrosis, suggesting them as potential candidates in the treatment of organ fibrosis.

However, some limitations hinder the recognition and extensive use of SUMOylation/NEDDylation activators and inhibitors in the treatment of organ fibrosis. The primary drawback is the limited number of clinical and preclinical investigations on SUMOylation/NEDDylation activators and inhibitors, especially the lack of high-quality evidence identifying their therapeutic effects and mechanisms. Few clinical studies have shown the therapeutic effects and mechanisms of the SUMOylation and NEDDylation activators and inhibitors in the treatment of organ fibrosis. Notably, some clinical trials have been designed to investigate the effects of MLN4924 in the treatment of advanced solid tumors, acute myeloid leukemia, and myelodysplastic syndromes (Adès et al., 2022; Saliba et al., 2023; Sarantopoulos et al., 2016; Short et al., 2023); the SAE inhibitor subasumstat (TAK-981) was designed to treat head and neck carcinomas (Derry et al., 2023). Although these clinical trials have not targeted organ fibrosis, they can provide references for their potential use in organ fibrosis. Another limitation is the identification of potentially efficient SUMOylation and NEDDylation substrates. For example, the roles of TGF-β and PML are vital in organ fibrosis, and their SUMOylation/NEDDylation are considered as important regulators of activity in organ fibrosis. Hence, efficient substrates need to be identified and verified, through which we could also obtain references beyond the research scope of organ fibrosis. We can use bioinformatics methods like feature extraction and machine learning (Zhao et al., 2022) to predict SUMOylation sites. The level of NEDD8 can be a potential marker of organ fibrosis. The Cancer Genome Atlas (TCGA) database and tissue arrays can be used to evaluate the clinical relevance of NEDD8 expression in disease, and quantitative proteomic analyses may be helpful for exploring the knockdown of disturbed biological pathways (Xian et al., 2021). Biotinylated NEDD8 (^bio^NEDD8) transgenic mice can be used in the pull-down of NEDDylated liver proteins and characterization of NEDDylomes in liver injury models (Serrano-Maciá et al., 2023) as promising strategies for fast selection and identification of NEDDylated proteins. Additionally, the use of new methods in cancer research allows SUMOylation-related genes as potential novel prognostic signatures and predictors of organ fibrosis (Sun et al., 2023; Wang et al., 2023a). Although clinical and related studies on SUMOylation and NEDDylation as well as their activators and inhibitors are limited, the potential of SUMOylation/NEDDylation has been verified in organ fibrosis treatment. Overall, SUMOylation and NEDDylation are promising therapeutic targets for organ fibrosis, so deeper investigations and clinical trials are needed to verify the therapeutic benefits of their activators and inhibitors to patients.
